# The Association between Diabetes-Related Distress and Medication Adherence in Adult Patients with Type 2 Diabetes Mellitus: A Cross-Sectional Study

**DOI:** 10.1155/2020/4760624

**Published:** 2020-03-01

**Authors:** Irene A. Kretchy, Augustina Koduah, Thelma Ohene-Agyei, Vincent Boima, Bernard Appiah

**Affiliations:** ^1^Department of Pharmacy Practice and Clinical Pharmacy, School of Pharmacy, College of Health Sciences, University of Ghana, P.O. Box LG 43, Legon, Ghana; ^2^Department of Medicine and Therapeutics, School of Medicine and Dentistry, College of Health Sciences, University of Ghana, P.O. Box GP 4236, Accra, Ghana; ^3^Centre for Science and Health Communication, PMB M71, Ministries, Accra, Ghana; ^4^Department of Environmental and Occupational Health, School of Public Health, Texas A&M University Health Science Center, 212 Adriance Lab Rd, 1266 TAMU, College Station, Texas, USA

## Abstract

**Background:**

Type 2 diabetes mellitus (T2DM) is a major public health problem associated with distress. T2DM can affect health outcomes and adherence to medications. Little is however known about the association between diabetes distress and medication adherence among patients with T2DM in Ghana.

**Objective:**

The objective of the present study is twofold: to estimate distress associated with T2DM and to examine its association with medication adherence.

**Methods:**

A hospital-based cross-sectional study was conducted among 188 patients with T2DM recruited from a diabetes specialist outpatient clinic at the Pantang Hospital in Accra, Ghana. Data were obtained using the Problem Areas In Diabetes (PAID) scale and the Medication Adherence Report Scale.

**Results:**

The findings showed that about 44.7% of the patients showed high levels of diabetes-related distress. Poor adherence to medications was recorded in 66.5% of the patients. Patients who were highly distressed had 68% lower odds of adhering to their medications compared to those who were not (OR: 0.32, 95% CI: 0.15-0.65). A principal component analysis revealed four areas of T2DM distress which were conceptualized as negative emotions about diabetes, dietary concerns and diabetes care, dissatisfaction with external support, and diabetes management helplessness.

**Conclusion:**

Our findings suggest that diabetes distress is a significant determinant of medication adherence behaviour in patients with T2DM. Thus, incorporating routine screening for distress into the standard diabetes care within the Ghanaian health system and having health practitioners adopt holistic approaches to diabetes management will be important context-specific interventions to improve adherence and health outcomes of people living and coping with T2DM.

## 1. Introduction

Type 2 diabetes mellitus (T2DM) is a group of metabolic diseases characterised by elevated levels of blood glucose, leading to serious damage to other organs over time [1]. It is a major cause of morbidity, disability, and mortality among populations worldwide [2], and it is associated with increasing unhealthy lifestyle such as poor dietary choices, lack of exercise, and inadequate physical activity [3, 4]. An estimated global prevalence of T2DM for age groups 20-79 was 8.8% in 2015, and it is expected to increase to 10.4% of adults by 2040 [5]. A greater burden of 75% of persons with diabetes is in developing countries [5]. The prevalence of T2DM in Ghana is reported to be 6.46%, and this is expected to rise by 2040 [5, 6].

Type 2 diabetes mellitus is associated with negative emotions such as anxiety, depression, and distress, and these emotions have been associated with poor clinical consequences including medication nonadherence [7–10] and glycemic outcomes [8, 11, 12]. Diabetes distress reflects negative feelings surrounding the disease and refers to the emotional response to the struggles, concerns, and worries associated with the broader demands of diabetes [13]. With time, diabetes distress becomes part of the diabetes experience for many patients and it is usually context-specific [14]. The distress is from the daily hassles and demands of the disease management [15], worries about poor glycemic control [16], fears about diabetic complications [16], poor support from significant others [17, 18], stigma [19], and financial difficulties [20]. When diabetes distress becomes protracted and is not identified and managed, patients experience burnout resulting in feelings of helplessness, hopelessness, and frustration with T2DM care [21–23]. Burnout may sometimes be physiologically triggered following an acute hyperglycemic crisis [24]. Patients with high diabetes-related distress are likely to demonstrate poor self-management [21].

A systematic review of studies on T2DM in sub-Saharan Africa reported a lack of studies on the psychosocial aspect of diabetes management [25]. In Ghana, despite the presence of some policy drive and programmatic responses to T2DM in particular and chronic noncommunicable diseases in general, the effect of these programmes is yet to reflect in the lives of patients [26, 27]. Similar to other sub-Saharan African countries, the psychosocial dimensions of the illness experience have been largely unexplored in Ghana [28, 29]. Previous studies on diabetes in Ghana have focused primarily on the prevalence and determinants [6, 30–34]. The primary objective of the study therefore was to estimate diabetes-specific distress and assess its impact on optimising treatment and adherence to medication in patients with T2DM. Further, the study explored the dimensions of diabetes-specific distress using principal component analysis of the Problem Areas In Diabetes (PAID) scale to observe variations and emphasize patterns of distress in the patients [7]. Although the associations between diabetes distress and adherence have been assessed previously in other countries [8–12], such studies have not been done in Ghana. This has created a gap in knowledge in the context of T2DM from an Afrocentric perspective. While medications for managing T2DM in Ghana are readily available, adherence to these medications is still not optimal [35, 36]. Thus, the information from this study will facilitate context-specific understanding of the problem from a psychosocial perspective so that culturally appropriate adherence solutions can be instituted.

## 2. Methods

### 2.1. Study Design

This was a hospital-based cross-sectional study of patients with T2DM who had reported for a clinical review at the outpatient clinic at the Pantang Hospital in Accra, Ghana. The facility attends to an average of 35 patients with diabetes in a week with approximately 3 new cases reporting within the week.

### 2.2. Participants

A total of 188 patients with T2DM (91.3%) were part of this study from the Pantang Hospital between May and July 2017, out of the 206 eligible participants using a simple random sampling method. The minimum sample size of 105 was obtained using the formula by Cochran (1963) and the estimated prevalence of diabetes in Ghana at 6.46% [6]:

(1) $$\begin{array}{c} n_{0}=\left(\text{Deff}\right)\frac{{\left(Z_{\propto /2}\right)}^{2}p\left(1-p\right)}{e^{2}}, \end{array}$$

where *n*_0_ is the minimum sample size, *Z*_∝/2_ is 1.96 at a confidence interval at 95%, *e* is the level of precision, *p* is the estimated proportion of patients with T2DM, and Deff is the design effect set at 1.03 and assuming a 10% nonresponse rate.

The study participants were adult patients with T2DM aged 18 years and over. Participants with type 1 diabetes, gestational diabetes, maturity-onset diabetes of the young, or latent autoimmune diabetes in adults were excluded from the study. Participants were also excluded if they had been diagnosed of any known psychiatric disorder according to their medical records. The hospital attends to patients with mental and physical conditions, and this exclusion criterion was necessary to avoid any known mental illness from being a possible confounder.

### 2.3. Measures

All participants completed questions on demographic characteristics with other clinical data about blood glucose levels obtained from the patient records. Diabetes distress was assessed using the Problem Areas In Diabetes Questionnaire [37]. This is a 20-item measure describing negative emotions related to T2DM such as anger, fear, and frustration. It uses a 5-point Likert response scale from 0 representing “no problem” to 4 representing “serious problem.” To obtain the total scores ranging from 0 to 100, initial scores are multiplied by 1.25. Higher scores indicate greater distress with T2DM. While participants with scores of 40 and above were highly distressed, very low scores of 0–10 may be indicative of patients in denial. The measure of reliability using Cronbach's alpha for the PAID scale in this study was 0.8299.

To estimate the level of adherence to medications, the study used the Medication Adherence Report Scale (MARS) which assesses both intentional and unintentional nonadherence to medicines [38]. Participants responded to five items (e.g., I forget to take my medicines) on a 5-point scale from “always” to “never,” and a total score of 25 and more indicated better adherence to medications [38]. The MARS has been previously used for patients with T2DM in Singapore [39]. In this study, Cronbach's alpha was 0.6967.

### 2.4. Ethical Consideration

The study received ethical approval from the Ethical Review Committee of the Ghana Health Service with approval number GHS-ERC:130/12/17. Permission was also obtained from the Administrator of the Pantang Hospital to conduct the study in the facility.  Informed consent was obtained from the sampled diabetic patients, and confidentiality/privacy was assured before their participation in the study.

### 2.5. Data Analysis

STATA version 14 was used for the data analysis. Frequencies and percentages were reported as descriptive statistics for categorical variables. Principal component analysis with varimax rotation analysis was used to explore diabetes distress from the various elements of the PAID scale. For continuous variables, means with standard deviations were reported for normally distributed data while median and interquartile ranges were reported as descriptive statistics when the normality assumption was violated. Normality assumption of continuous variables was tested with the skewness and kurtosis Shapiro-Francia tests. Chi-squared and Fisher exact tests of independence were used to test for association between categorical independent variables and the outcome variables (diabetes distress and medication adherence). The Wilcoxon rank sum test was used to compare the median of glucose levels across the various categories of the outcome variables. Binary logistic regression models were used to determine the effects of the independent variables on the outcome variables. In assessing the association between the individual item responses of PAID and medication adherence, the item responses were dichotomized into patients who regarded the item as “a problem” and “not a problem” [16, 40]. The statistical test of significance was set at 5%.

## 3. Results

### 3.1. Patient Characteristics

Table 1 shows the background and clinical characteristics of the patients with T2DM (*n* = 188). On average, the patients were 59.3 ± 11.9 years old, with females constituting the majority (72.3%). Fifty-nine percent of the patients had at least one comorbidity and were prescribed a mean of 2.6 ± 1.1 medications for their conditions. Averagely, participants had a glucose level of less than or equal to 7.8 (IQR: 6.4–11.0).

### 3.2. Diabetes Distress

While the average PAID score among the patients was 36.0 ± 4.3, 44.7% (84/188) showed high levels of distress with PAID scores ≥ 40. The chi-squared test showed significant associations between comorbidities (*p* = 0.049), glucose levels (0.006), and high distress. However, results from the multiple logistic regression model showed that the blood glucose level was the only significant predictor of distress (*p* < 0.001). The odds of high diabetic distress among patients with T2DM increased by 12% with every additional unit increase in the glucose level (OR: 1.12, 95% CI: 1.04-1.21) (Table 2).

### 3.3. Medication Adherence

The average MARS-5 score was 21.2 ± 3.6, with one-third of the patients optimally adhering to their medications. The blood glucose level was not significantly associated with medication adherence, and higher levels were observed among patients with poor medication adherence compared with those who adhered completely to their medications (8.4 vs. 7.1, *p* = 0.056).

### 3.4. High Diabetes Distress and Medication Adherence

The proportion of patients with T2DM who exhibited high distress had significantly lower scores on medication adherence compared with those who were not distressed (20.2 vs. 44.2, *p* = 0.001). Those with high distress had 68% lower odds of adhering to their medications compared to those who were not distressed (OR: 0.32, 95% CI: 0.15-0.65) (Table 3). Ten out of the twenty items on diabetes distress using the PAID scale showed significant associations with medication adherence (*p* < 0.05) (Table 4). These included discouragement with diabetes treatment, uncomfortable social situations, and feelings of anger, anxiety, guilt, loneliness, and burnout.

### 3.5. Components of Diabetes Distress

Table 5 shows the principal component analysis of four components with eigenvalues exceeding 1, explaining 27.18%, 7.49%, 6.91%, and 6.43% of the variance, respectively.

Factor 1 was conceptualized as negative emotions about diabetes consisting of thirteen variable loadings of items such as the following: “Scared about thoughts of living with diabetes” (0.643), “Depressed about thoughts of diabetes” (0.708), “Worry about the future and complications” (0.610), “Guilt and anxiety when off-track management of diabetes” (0.603), and “Diabetes taking too much of mental energy” (0.540). The second factor was noted as dietary concerns and diabetes care consisting of two variable loadings: “Feelings of deprivation regarding food and meals” (0.596) and “No clear or concrete goals for diabetes care” (0.529). Factor 3 was referred to as dissatisfaction with external support. This comprised two variable loadings: “Feeling unsatisfied with diabetes physician” (0.464) and “Friends and family not supportive” (0.596). Diabetes management helplessness was the fourth factor, constituted by two variables: “Coping with complications of diabetes” and “Feeling burned out by constant effort to manage diabetes.” The two variables had 0.510 and 0.489 varimax rotational loadings, respectively.

## 4. Discussions

The present study contributes to knowledge of illness experience of patients with T2DM from a psychosocial perspective with emphasis on diabetes distress. While studies on diabetes distress have mainly been from developed countries, fewer studies have emerged from Africa [25]. This study therefore focused on patients with T2DM in a Ghanaian health context, bearing in mind the cultural influences of illness experiences on health outcomes.

In this study, patients who experienced diabetes distress tended to poorly adhere to their medications compared with those who were not distressed about their disease experience and outcome. Diabetes distress is an essential predictor of clinical outcomes in T2DM care, and it has been linked to poor self-management, treatment adherence, and blood glucose status in such patients [41].

Findings from the PCA further showed four areas of distress by patients with T2DM. These are negative emotions, dietary concerns, dissatisfaction with external support, and diabetes management helplessness. The concept of diabetes distress in this context could be suggested to mean a combination of these four areas of concern. In a related study where distress was assessed using the diabetes distress scale, a different measure from what was used in this study, PCA yielded factors that were similar to what was obtained in this study [42]. Yet in another study involving southern rural African-American patients with T2DM, a PCA of the PAID yielded two factors: lack of confidence and negative emotional consequences [7].

Living with T2DM can negatively impact on the psychological well-being of patients. Previous studies on negative emotions with its affective correlates in T2DM have primarily focused on depression, mainly based on clinical presentations and diagnosis in the *Diagnostic and Statistical Manual for Mental Disorders* [43–45]. Yet, the prospective effect of negative emotions with subclinical symptoms also has clinical implications for T2DM care and adherence [46].

Emotionally negative symptoms like discouragement, anger, anxiety, guilt, and burnout have been indicated as risk factors for physical health and health-related outcomes including medication adherence [16, 47]. This corroborates our findings from the item analysis of the PAID and adherence. Due to the chronicity of T2DM and the fact that patients with T2DM will have to manage with living with the condition for the rest of their lives, our findings suggest the need for interventions targeting positive emotions which can buffer the effects of the negative emotions. This is because positive feelings are important in chronic diabetes care and outcomes [48]. In a related study, a meaningful relationship between the levels of positive affect and adherence measures was reported [41]. Thus, support services for T2DM can address potentially harmful effects of negative feelings and emphasize benefits of positive emotionality for better health-related outcomes. Such support services could include the use of culturally relevant communication interventions including mobile phones, mass media, social media, and face-to-face approaches and social media to address emotional needs of patients with T2DM experiencing distress.

The study also showed that T2DM patients were distressed about the nature of care from their physicians and support from social relations. In addition, having uncomfortable social situations and the feeling of loneliness with T2DM were significantly associated with poor medication adherence. This finding indicates that, from the perspective of the patients, living with T2DM could be synonymous with feeling alone with poor social support from significant others, regardless of evidence that psychosocial support is helpful in adaptive behaviours by patients with T2DM [49]. Support and care from the social networks of patients including health care professionals, family, friends, neighbours, colleagues, and fellow patients with T2DM could help them take positive stances, build resilience, relieve distress, and improve on their well-being [50, 51]. Furthermore, care and support from formal and familial contacts have positive effects on the medication adherence behaviour in patients with T2DM due to the encouragement of optimism in such patients [52].

The prevalence of both chronic microvascular and acute complications of T2DM is much greater in patients with poor glycemic control and poor dietary quality [10, 53, 54]. Concerns about meeting dietary requirements and following treatment plans were also synonymous to the concept of diabetes distress in this study. Psychological distress has been associated with a high risk of T2DM complications, and in this study, patients who had difficulty dealing with their complications reported poor medication adherence behaviour. The occurrence of diabetic complications has been proposed to be significantly higher in nonadherent patients compared to those who adhere to medications [53].

As indicated in Introduction, patients with high diabetes-related distress show signs of poor self-management of T2DM [21], and in this study, high blood glucose levels and distress were significantly related. Similar to previous studies, this study suggests that assessment and management of distress in patients with T2DM are crucial in determining health outcomes. It was observed that participants who felt highly distressed by the constant effort needed to manage T2DM poorly adhered to their medications [55, 56].

This study has some limitations. First, a mixed method design could have been adopted for this study in order to explore the concept of diabetes distress from a more qualitative approach. Second, respondents are from only one hospital; thus, the findings may not be representative of the general population patients with T2DM in Ghana; in addition, causality could not be established nor could the direction of the effect of diabetes distress on adherence be determined because the data were obtained through a cross-sectional study approach. Given that this study relied on self-reports, recall bias could be a limitation. In spite of these limitations, this study is among the first to report the association between T2DM distress and nonadherence in Ghana, thus identifying areas of context-specific interventions to improve adherence in patients with T2DM.

### 4.1. Implications for Clinical Practice

These study findings are significant in explaining the association between psychosocial interactions and health outcomes in patients with T2DM. Based on the results, psychological and social context-specific interventions that address diabetes distress should be considered when patients with diabetes are managed at health institutions. For example, culturally relevant communications may need to be developed, pilot-tested, and implemented to address psychological issues confronting patients with T2DM in Ghana. Other cultural dimensions that impact treatment outcomes such as religiosity could also be explored in the management of T2DM in Ghana.

### 4.2. Implications for Policy

It may also be relevant to recommend that routine screening for diabetes distress be incorporated into national standard diabetes treatment guidelines for care within the health care systems in Ghana. This is to ensure that patients with a possible risk of distress can receive more comprehensive diabetic care from a psychosocial perspective.

## 5. Conclusion

This study investigated the link between diabetes distress and medication adherence in patients with T2DM in Ghana. The information suggests that diabetes distress is a significant determinant of medication adherence behaviour. Thus, incorporating routine screening for distress into the standard diabetes care within the Ghanaian health system and having health practitioners adopt a biopsychosocial approach to diabetes management will be important context-specific interventions to improve health outcomes of people living and coping with T2DM.

## Figures and Tables

**Table 1:** Background and clinical characteristics of patients with T2DM receiving treatment at Pantang Hospital.

	Frequency	Percent
Age		
Mean ± SD	59.31 ± 11.94	
≤50	41	21.81
51-60	62	32.98
61+	85	45.21
Sex		
Female	136	72.34
Male	52	27.66
Glucose: median (LQ, UQ)	7.8 (6.4, 11)	
Comorbidity		
No	77	40.96
Yes	111	59.04
Number of medications		
Mean ± SD	2.56 ± 1.13	
One	31	16.58
Two	69	36.90
Three	54	28.88
Four or more	33	17.65
PAID score		
Mean ± SD	36.03 ± 4.28	
Not highly distressed (PAID score < 40)	104	55.32
Highly distressed (PAID score ≥ 40)	84	44.68
MARS-5 score		
Mean ± SD	21.23 ± 3.64	
Low medication adherence (MARS score < 25)	125	66.49
Medication adherent (MARS score of 25)	63	33.51

SD: standard deviation; LQ: lower quartile; UQ: upper quartile.

**Table 2:** Association between background and clinical characteristics and high distress in patients with T2DM receiving treatment at Pantang Hospital.

	High distress		Chi-square	*p* value	Adjusted logistic regression model	
	No, *n* (%)	Yes, *n* (%)			Odds ratio	*p* value
Sex			0.01	0.939		0.485
Female	75 (55.15)	61 (44.85)			ref	
Male	29 (55.77)	23 (44.23)			0.78 (0.38–1.57)	
Age			2.23	0.327		0.943
≤50	20 (48.78)	21 (51.22)			ref	
51-60	32 (51.61)	30 (48.39)			0.98 (0.42–2.33)	
61±	52 (61.18)	33 (38.82)			0.89 (0.38–2.05)	
Glucose level: median (LQ, UQ)	7.3 (6.2, 10)	9.2 (6.7, 12.5)		0.006^∗∗^^§^	1.12 (1.04–1.21)	<0.001^∗∗∗^
Number of medications			4.75	0.191		0.138
One	18 (58.06)	13 (41.94)			ref	
Two	31 (44.93)	38 (55.07)			2.35 (0.92–6.03)	
Three	34 (62.96)	20 (37.04)			1.06 (0.38–2.92)	
Four or more	20 (60.61)	13 (39.39)			1.38 (0.44–4.30)	
Comorbidity			3.87	0.049		0.130
No	36 (46.75)	41 (53.25)			ref	
Yes	68 (61.26)	43 (38.74)			0.58 (0.29–1.16)	

%: row percentages; *n*: number of observations; ^∗^*p* < 0.01, ^∗∗^*p* < 0.01, and ^∗∗∗^*p* < 0.001; CI: confidence interval; ref: reference category; LQ: lower quartile; UQ: upper quartile, §: *p* value obtained from a Wilcoxon rank sum test.

**Table 3:** Association between background and clinical characteristics and medication adherence status of patients with T2DM receiving treatment at Pantang Hospital.

	Adherent (MARS-5 score of 25)		Chi-square	*p* value	Adjusted logistic regression model	
	No, *n* (%)	Yes, *n* (%)			Odds ratio	*p* value
Sex			0.3	0.587		0.370
Female	92 (67.65)	44 (32.35)			ref	
Male	33 (63.46)	19 (36.54)			1.41 (0.67–2.98)	
Age			1.19	0.551		0.422
≤50	30 (73.17)	11 (26.83)			ref	
51-60	39 (62.9)	23 (37.1)			1.86 (0.73–4.75)	
61±	56 (65.88)	29 (34.12)			1.41 (0.56–3.52)	
Glucose level: median (LQ, UQ)	8.4 (6.6, 11.3)	7.1 (6.2, 10.5)		0.056^§^	0.98 (0.91–1.07)	0.721
Number of medications			5.79	0.122		0.161
One	19 (61.29)	12 (38.71)			ref	
Two	51 (73.91)	18 (26.09)			0.66 (0.24–1.78)	
Three	38 (70.37)	16 (29.63)			0.71 (0.25–2.00)	
Four or more	17 (51.52)	16 (48.48)			1.83 (0.58–5.81)	
Comorbidity			0.01	0.951		0.471
No	51 (66.23)	26 (33.77)			ref	
Yes	74 (66.67)	37 (33.33)			0.76 (0.36–1.61)	
High distress			12.01	0.001^∗∗^		0.002^∗∗^
No	58 (55.77)	46 (44.23)			ref	
Yes	67 (79.76)	17 (20.24)			0.32 (0.15–0.65)	

%: row percentages; *n*: number of observations; ^∗^*p* < 0.01, ^∗∗^*p* < 0.01, and ^∗∗∗^*p* < 0.001; CI: confidence interval; ref: reference category; LQ: lower quartile; UQ: upper quartile; §: *p* value obtained from a Wilcoxon rank sum test.

**Table 4:** Association between individual item responses of PAID scale and medication adherence status of patients with T2DM receiving treatment at Pantang Hospital.

Items	Total	Adherent (MARS-5 score of 25)		Unadjusted	
	*n* (%^a^)	No, *n* (%^b^)	Yes, *n* (%^b^)	Odds ratio	*p* value
No clear or concrete goals for diabetes care					0.468
No problem	137 (72.87)	89 (64.96)	48 (35.04)	ref	
Problem	51 (27.13)	36 (70.59)	15 (29.41)	0.77 (0.38–1.55)	
Feeling discouraged with diabetes treatment plan					0.007^∗∗^
No problem	108 (57.45)	63 (58.33)	45 (41.67)	ref	
Problem	80 (42.55)	62 (77.5)	18 (22.5)	0.41 (0.21–0.78)	
Scared about thoughts of living with diabetes					0.134
No problem	61 (32.45)	36 (59.02)	25 (40.98)	ref	
Problem	127 (67.55)	89 (70.08)	38 (29.92)	0.61 (0.33–1.16)	
Uncomfortable social situations related to diabetes					0.002^∗∗^
No problem	75 (39.89)	40 (53.33)	35 (46.67)	ref	
Problem	113 (60.11)	85 (75.22)	28 (24.78)	0.38 (0.2–0.7)	
Feelings of deprivation regarding food and meals					0.104
No problem	25 (13.3)	13 (52)	12 (48)	ref	
Problem	163 (86.7)	112 (68.71)	51 (31.29)	0.49 (0.21–1.16)	
Feeling depressed about thoughts of diabetes					0.313
No problem	51 (27.13)	31 (60.78)	20 (39.22)	ref	
Problem	137 (72.87)	94 (68.61)	43 (31.39)	0.71 (0.36–1.38)	
Not knowing if mood is related to diabetes					0.009^∗∗^
No problem	68 (36.17)	37 (54.41)	31 (45.59)	ref	
Problem	120 (63.83)	88 (73.33)	32 (26.67)	0.43 (0.23–0.81)	
Feeling overwhelmed by diabetes					0.177
No problem	40 (21.28)	23 (57.5)	17 (42.5)	ref	
Problem	148 (78.72)	102 (68.92)	46 (31.08)	0.61 (0.3–1.25)	
Worrying about low sugar reactions					0.019^∗^
No problem	57 (30.32)	45 (78.95)	12 (21.05)	ref	
Problem	131 (69.68)	80 (61.07)	51 (38.93)	2.39 (1.16–4.95)	
Feeling angry about thought of living with diabetes					0.023^∗^
No problem	60 (31.91)	33 (55)	27 (45)	ref	
Problem	128 (68.09)	92 (71.88)	36 (28.13)	0.48 (0.25–0.91)	
Feeling constantly concerned about food and eating					0.696
No problem	13 (6.91)	8 (61.54)	5 (38.46)	ref	
Problem	175 (93.09)	117 (66.86)	58 (33.14)	0.79 (0.25–2.53)	
Worrying about the future and complications					0.100
No problem	30 (15.96)	16 (53.33)	14 (46.67)	ref	
Problem	158 (84.04)	109 (68.99)	49 (31.01)	0.51 (0.23–1.13)	
Guilty and anxious when off track management					0.022^∗^
No problem	49 (26.06)	26 (53.06)	23 (46.94)	ref	
Problem	139 (73.94)	99 (71.22)	40 (28.78)	0.46 (0.23–0.89)	
Not accepting “diabetes”					0.085
No problem	115 (61.17)	71 (61.74)	44 (38.26)	ref	
Problem	73 (38.83)	54 (73.97)	19 (26.03)	0.57 (0.3–1.08)	
Feeling unsatisfied with diabetes physician					0.089
No problem	131 (69.68)	82 (62.6)	49 (37.4)	ref	
Problem	57 (30.32)	43 (75.44)	14 (24.56)	0.54 (0.27–1.1)	
Diabetes taking too much of mental energy					<0.001^∗∗∗^
No problem	50 (26.6)	21 (42)	29 (58.00)	ref	
Problem	138 (73.4)	104 (75.36)	34 (24.64)	0.24 (0.12–0.47)	
Feeling alone with diabetes					<0.001^∗∗∗^
No problem	84 (44.68)	44 (52.38)	40 (47.62)	ref	
Problem	104 (55.32)	81 (77.88)	23 (22.12)	Six	
Friends and family not supportive					0.497
No problem	69 (36.7)	48 (69.57)	21 (30.43)	ref	
Problem	119 (63.3)	77 (64.71)	42 (35.29)	1.25 (0.66-2.35)	
Coping with complications of diabetes					<0.001^∗∗∗^
No problem	76 (40.43)	39 (51.32)	37 (48.68)	ref	
Problem	112 (59.57)	86 (76.79)	26 (23.21)	0.32 (0.17-0.6)	
Feeling “burned out” by constant effort to manage					0.008^∗∗^
No problem	65 (34.57)	35 (53.85)	30 (46.15)	ref	
Problem	123 (65.43)	90 (73.17)	33 (26.83)	0.43 (0.23-0.8)	

%^a^: column percentage; %^b^: row percentages; *n*: number of observations; ^∗^*p* < 0.01, ^∗∗^*p* < 0.01, and ^∗∗∗^*p* < 0.001; CI: confidence interval; ref: reference category; LQ: lower quartile; UQ: upper quartile; *p* values were obtained from an unadjusted binary logistic regression model.

**Table 5:** Factor loadings, communalities (*h*^2^), and percent of variance for principal factor extraction and varimax rotation on items of diabetes distress.

Items	^a^ *F*_1_	*F*_2_	*F*_3_	*F*_4_	*h* ^2^
Negative emotions about diabetes					
Feeling discouraged with diabetes treatment plan	0.472				0.344
Scared about thoughts of living with diabetes	0.643				0.604
Uncomfortable social situations related to diabetes	0.640				0.495
Feeling depressed about thoughts of diabetes	0.708				0.689
Not knowing if mood is related to diabetes	0.709				0.597
Feeling overwhelmed by diabetes	0.466				0.494
Feeling angry about thought of living with diabetes	0.606				0.656
Worrying about the future and complications	0.610				0.519
Guilty and anxious when off-track management	0.603				0.435
Diabetes taking too much of mental energy	0.540				0.500
Feeling alone with diabetes	0.570				0.528
Coping with complications of diabetes	0.603				0.712
Feeling burned out	0.611				0.641
Dietary concerns and diabetes care					
No clear or concrete goals for diabetes care		0.529			0.711
Feelings of deprivation regarding food and meals		0.596			0.600
Dissatisfaction with external support					
Feeling unsatisfied with diabetes physician			0.464		0.736
Friends and family not supportive			0.596		0.627
Diabetes management helplessness					
Coping with complications of diabetes				0.510	0.712
Feeling burned out by constant effort to manage diabetes				0.489	0.641
Percent variance	27.179	7.485	6.906	6.427	

^a^Factor labels: *F*_1_: negative emotions about diabetes; *F*_2_: dietary concerns and diabetes care; *F*_3_: dissatisfaction with external support; *F*_4_: diabetes management helplessness.

## Data Availability

The data that support the findings of this study are available from the corresponding author, [IAK], upon reasonable request.
